# *Salmonella* Phage ST64B Encodes a Member of the SseK/NleB Effector Family

**DOI:** 10.1371/journal.pone.0017824

**Published:** 2011-03-18

**Authors:** Nat F. Brown, Brian K. Coombes, Jenna L. Bishop, Mark E. Wickham, Michael J. Lowden, Ohad Gal-Mor, David L. Goode, Erin C. Boyle, Kristy L. Sanderson, B. Brett Finlay

**Affiliations:** 1 Institute for Glycomics, Griffith University, Gold Coast, Queensland, Australia; 2 Michael Smith Laboratories, University of British Columbia, Vancouver, British Columbia, Canada; Indian Institute of Science, India

## Abstract

*Salmonella enterica* is a species of bacteria that is a major cause of enteritis across the globe, while certain serovars cause typhoid, a more serious disease associated with a significant mortality rate. Type III secreted effectors are major contributors to the pathogenesis of *Salmonella* infections. Genes encoding effectors are acquired *via* horizontal gene transfer, and a subset are encoded within active phage lysogens. Because the acquisition of effectors is in flux, the complement of effectors possessed by various *Salmonella* strains frequently differs. By comparing the genome sequences of *S. enterica* serovar Typhimurium strain SL1344 with LT2, we identified a gene with significant similarity to SseK/NleB type III secreted effector proteins within a phage ST64B lysogen that is absent from LT2. We have named this gene *sseK3*. SseK3 was co-regulated with the SPI-2 type III secretion system *in vitro* and inside host cells, and was also injected into infected host cells. While no role for SseK3 in virulence could be identified, a role for the other family members in murine typhoid was found. SseK3 and other phage-encoded effectors were found to have a significant but sparse distribution in the available *Salmonella* genome sequences, indicating the potential for more uncharacterised effectors to be present in less studied serovars. These phage-encoded effectors may be principle subjects of contemporary selective processes shaping *Salmonella*-host interactions.

## Introduction

*Salmonella enterica* is a Gram-negative intracellular bacterial pathogen of animals. *Salmonella* is a major cause of enteritis across the world and is also the cause of typhoid, a frequently fatal disease prevalent in developing nations. The pathogenesis of *Salmonella* induced enteritis involves penetration of the intestinal epithelium and, through interactions with epithelial and immune cells, *Salmonella* induces significant inflammation in the intestine resulting in diarrhoea [1]. During typhoid, following penetration of the intestinal epithelium *Salmonella* enters phagocytic cells and is transported to the mesenteric lymph nodes and to the liver and spleen [1]. At these sites *Salmonella* resides primarily within macrophages and initiates substantial inflammation [2], [3].

The principle factors allowing *Salmonella* to penetrate the intestinal epithelium and replicate within phagocytes of the host are ‘effectors’, proteins that are injected into infected host cells by type III secretion systems (T3SSs) [4]. *Salmonella* encodes two separate T3SSs, each of which is generally considered to function at distinct stages of pathogenesis. T3SS-1 and T3SS-2 (encoded by Salmonella pathogenicity islands 1 and 2, respectively) translocate effectors that collectively mediate penetration of the intestinal epithelium (T3SS-1) [5] or replication within phagocytic cells (T3SS-2) [6], [7]. In the host cell, effectors are hypothesized to interact with specific host molecules, which are subverted in order to benefit the infecting *Salmonella* [1].

While *Salmonella* encodes two separate T3SSs, each of which was acquired *via* horizontal gene transfer, the genes encoding effectors are typically encoded at unlinked genomic sites and were likely horizontally acquired separately from SPI1 and 2. Commensurate with the theme of frequent horizontal acquisition of effector-encoding genes, different strains of *Salmonella* commonly possess different arsenals of effectors. Even the closely related *S. enterica* serovar Typhimurium (*S. typhimurium*) strains SL1344 and 14028, which are the most commonly used model strains used in pathogenesis studies, possess different complements of effectors. SL1344 possesses *sopE* whilst 14028 does not, and *vice versa* for *sspH1* [8]. Each of these effectors is encoded on a separate prophage (SopEϕ and Gifsy-3, respectively) [8], [9].

To date, the only published and annotated genome sequence of a *S. typhimurium* strain is that of LT2, an avirulent strain that serves primarily as a model for bacterial and phage genetics. The genome sequence of the virulent strain SL1344 has recently been completed and is currently being annotated at the Wellcome Trust Sanger Institute. Relying on the hypothesis that the genetic complement of effectors frequently varies between strains, we aimed to identify novel effectors based on their similarity to known effectors by comparing the unannotated genome sequence of SL1344 to the LT2 genome. Based on this, we identified a novel member of the SseK/NleB family of effectors, which we have termed SseK3.

## Results

### Comparison of the SL1344 and LT2 genomes

Regions of the SL1344 genome that are not present in the genome of LT2 were identified by comparison using MEGABLAST and ARTEMIS COMPARISON TOOL as described in the materials and methods. This identified nine regions in the SL1344 chromosome that were not present in the LT2 chromosome, whereas the virulence plasmids were not significantly different. The nine unique regions of the SL1344 chromosome encoded 111 open reading frames (ORFs) from the preliminary annotation provided by the Sanger Institute, plus 6 additional ORFs we annotated ourselves. We performed BLAST searches of the nonredundant amino acid sequence database using the deduced amino acid sequence of each unique ORF as a query. We then analysed the output from each search with a focus on identifying unique ORFs with significant similarity to effectors.

**Figure 1:**
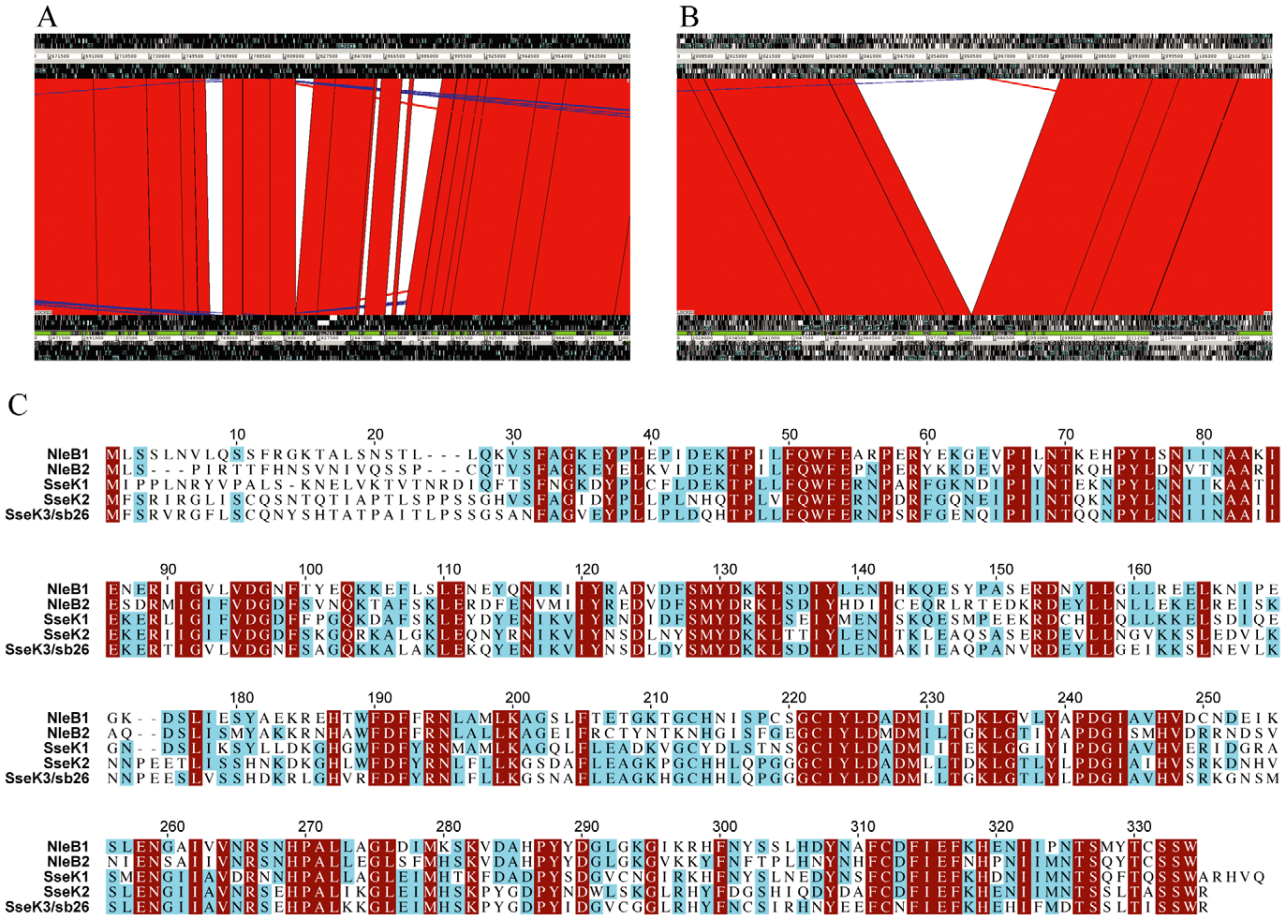
Phage encoded genomic differences between *S. typhimurium* SL1344 and LT2. A & B. Screenshots from ARTEMIS COMPARISON TOOL showing (A) differences in the SopEϕ/Fels-2 region and (B) the ST64B insertion in SL1344 relative to LT2. The SL1344 sequence is shown on top and LT2 at the bottom. The red regions indicate alignment between the two genome sequences and the white regions indicate gaps in the alignment. C. Alignment of the amino acid sequences of SseK3 and other SseK/NleB family members. Residues conserved in all five proteins are shaded in maroon, those conserved in three or four proteins are shaded in cyan. SseK1 and 2 sequences are from LT2 and NleB1 and 2 are from *E. coli* strain EDL933.

This analysis identified two genes with significant similarity to effectors. One of these, *sopE*, is known to be present in SL1344 [9] and absent from LT2 [10]. *sopE* is carried on a phage SopEϕ lysogen in SL1344 at the same locus as a phage Fels-2 lysogen in LT2. The phages SopEϕ and Fels-2 share a high degree of identity over most of their genomes, with numerous segments that appear to have been exchanged relative to each other that are represented in figure 1A [11]. One of these segments, unique to SL1344, encodes *sopE*.

The second gene encodes a protein that showed 75% identity to the effector SseK2 [12] from LT2 in a BLAST search. This gene corresponds to ORF sb26 encoded within a phage ST64B [13] lysogen carried by SL1344 [8], which is absent from LT2 [10]. Unlike the situation for *sopE*, where SL1344 and LT2 harbour distinct, but closely related lysogens at equivalent loci, the ST64B lysogen occupies an attachment site that is unoccupied in LT2 (Fig. 1B). As can be seen in the amino acid sequence alignment of the members of the SseK/NleB family of effectors, ORF sb26 clearly encodes for a protein that is highly related to this family of effectors (Fig. 1C). ORF sb26 is clearly distinct from the SL1344 orthologues of *sseK1* and *sseK2* from LT2, hence we propose that the ORF sb26 constitutes the third SseK/NleB family member identified within *Salmonella* and that it be renamed *sseK3*.

### Expression of SseK3

**Figure 2:**
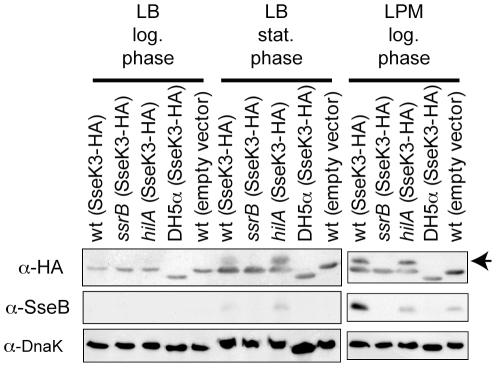
Expression of SseK3 in culture medium. Western blots of whole bacterial cell lysates from the culture conditions and strain genotypes indicated at the top of the figure were probed with the antibodies indicated to the left of the respective panels. The anti-HA panels show a cross-reacting band in each lane just below the SseK3 band. This cross-reacting band is lower in the *E. coli* lysates than the *Salmonella* lysates. The arrow to the right of the α-HA panel indicates the bands corresponding to SseK3-HA.

Regulation and expression of effectors is typically co-ordinated by pathogencity island encoded transcription factors that control expression of the respective T3SS. During culture *in vitro*, logarithmic phase growth in LB medium is most commonly used to induce expression of the SPI1 regulon, whereas logarithmic phase growth in low phosphate, low magnesium minimal (LPM) medium induces expression of the SPI2 regulon [14]–[16]. More recently, stationary phase growth in LB medium has been shown to also induce expression of the SPI2 regulon [17]. To search for any correlation of the expression pattern of SseK3 with the SPI1 and SPI2 regulons, a wild type *Salmonella* strain expressing HA epitope tagged SseK3 (SseK3-HA) from its native upstream region on a low copy number plasmid was cultured under the three culture conditions described above. Western blotting of whole cell lysates from these cultures and probing with anti-HA antibodies indicated that SseK3 is expressed in LPM medium and during stationary phase, but not logarithmic phase in LB medium (Fig. 2). Levels of the SPI2 protein, SseB, in the same samples showed a similar expression pattern as SseK3 (Fig. 2). As a further control, levels of the constitutively expressed protein, DnaK, were unaffected by growth condition (Fig. 2). Additionally, when *E. coli* DH5α transformed with the same SseK3 expression plasmid was subjected to the same experimental conditions, SseK3 expression could not be detected. This suggests that expression of SseK3 requires regulatory factors that are present in *Salmonella* and absent from *E. coli*.

Key transcription factors controlling expression of SPI1 and SPI2 genes are HilA and SsrB, respectively [6], [14], [16], [18]. Expression of SseK3 from *hilA* and *ssrB Salmonella* transformed with the same plasmid and under the same culture conditions as above was also analysed by western blotting. Mutation of *ssrB* completely abrogated expression of SseK3, whereas mutation of *hilA* had little affect (Fig. 2). This was paralleled in the SPI2 control protein, SseB, and neither mutation had any affect on expression of the constitutive control protein, DnaK (Fig. 2). These findings are consistent with SseK3 belonging to the SsrB (SPI2) regulon and not the HilA (SPI1) regulon.

**Figure 3:**
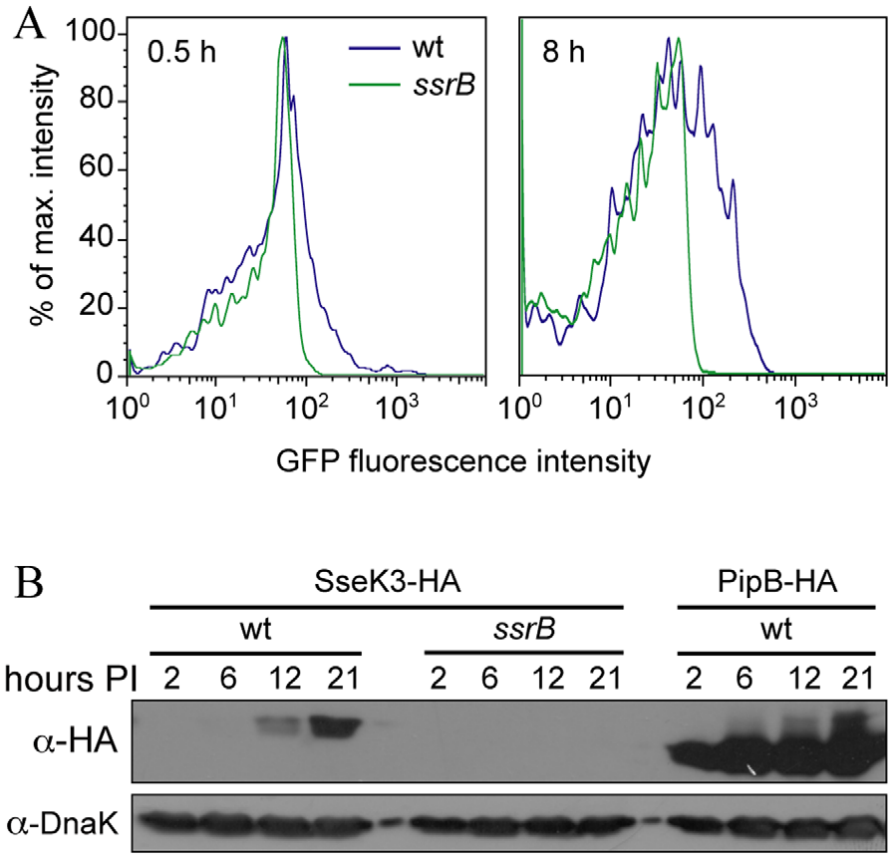
Expression of SseK3 in host cells. A. Graphs showing GFP fluorescence intensity of intracellular wild-type or *ssrB Salmonella* containing an *sseK3* promoter GFP fusion plasmid. Cells were infected for 0.5 h and 8 h before being processed for flow cytometry. B. Western blots of infected HeLa cell lysates made at the indicated times post-infection (hours PI), with the indicated strains transformed with plasmids encoding the indicated effector fused to tandem HA epitopes. The western blots were probed for the HA epitope and DnaK as a loading control.

A prominent feature of the SsrB regulon is induction of expression following entry of *Salmonella* into host cells [6]. If SseK3 belongs to the SsrB regulon its expression should also be induced within host cells. To test this hypothesis bone-marrow derived macrophages were infected with *Salmonella* transformed with a plasmid containing a transcriptional fusion of *gfp* to the *sseK3* upstream region, and at 0.5 and 8 h post-infection the *Salmonella* fluorescence intensity was quantified by flow cytometry. Transcription from the *sseK3* upstream region was induced in host cells, with higher levels observed at 8 h post-infection than 0.5 h (Fig. 3A). As a control, an *ssrB* strain transformed with the same plasmid showed little induction of SseK3 expression following uptake into host cells (Fig. 3A). As an alternative approach, and to show expression at the protein level in host cells, HeLa cells were infected for 2, 6, 12 and 21 h with wild type or *ssrB Salmonella* transformed with the SseK3-HA expression plasmid described above. Lysates from these infected cells, normalised to the number of *Salmonella*, were western blotted and probed for SseK3-HA expression, which could be observed to increase with time in the wild type, but not the *ssrB* strain (Fig. 3B). PipB is a SPI2 effector that belongs to the SsrB regulon [6], [19], and a control infection with wild type transformed with a PipB-HA expression plasmid also showed expression levels that increased with time during intracellular infection (Fig. 3B). These results show that SseK3 is expressed inside host cells in an SsrB dependent manner, and collectively demonstrate that SseK3 belongs to the SsrB regulon.

### SseK3 secretion and translocation into host cells

**Figure 4:**
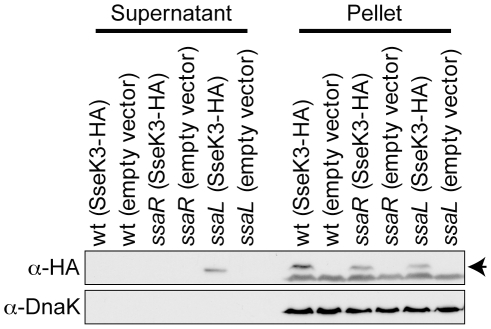
Secretion of SseK3. Western blots of concentrated culture supernatant proteins and whole *Salmonella* cell lysates (pellet) from strains genotypes indicated at the top of the figure were probed with the antibodies indicated to the left of the figure. The arrow to the right of the α-HA panel indicates the bands corresponding to SseK3-HA.

Secretion of most SPI2 effectors can be induced during *in vitro* culture in LPM medium at low pH (pH 5.8). Under such artificial conditions, T3SSs secrete distinct classes of substrates into the extracellular medium, the most abundant of which are effectors and translocators. It is hypothesised that secretion of these substrate classes is, at least in part, controlled by an InvE family member in each T3SS [20], [21], such that in an *invE* strain effectors are hypersecreted and translocators are not secreted [20]. The SPI2 InvE family member is SsaL, which when knocked out results in a lack of translocator secretion and hypersecretion of effectors into culture supernatants [22]. To investigate whether SseK3 is secreted in a manner consistent with being an effector, wild type, *ssaR* (T3SS-2 null) and *ssaL Salmonella* transformed with the HA tagged SseK3 expression plasmid were grown under secretion inducing conditions and culture supernatant proteins were analysed by western blotting. SseK3 was found in supernatants only from *ssaL* strain cultures (Fig. 4). Also, the constitutively expressed cytoplasmic control protein, DnaK, was detected in equal abundance and only associated with the bacterial pellet (Fig. 4). The lack of detectable secretion from wild type *Salmonella* is inconsistent with SseK3 being a secreted effector, although the low expression level of SseK3 may have precluded its detection. On the other hand, the increased level of SseK3 secretion from *ssaL Salmonella*, together with the demonstrated homology to SseK/NleB effectors (Fig. 1C) is consistent with SseK3 being a effector substrate of the T3SS-2 system.

**Figure 5:**
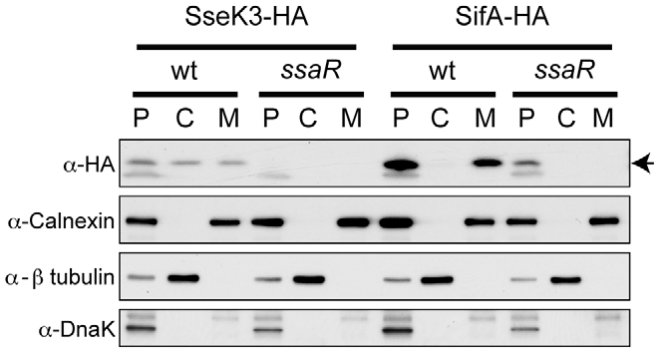
Translocation of SseK3 into infected HeLa cells. Western blots from fractionated, infected HeLa cells probed with the antibodies indicated to the left of the figure. SseK3-HA and SifA-HA indicated at the top of the figure refer to the HA-tagged protein expressed in the respective strain, and under this, wt and *ssaR* refer to the genotype of the respective strain. P, C and M refer to the fractions collected from the infected cells as described in the text. The arrow to the right of the α-HA panel indicates the bands corresponding to SseK3-HA and SifA-HA (which are approximately the same MW).

Ultimately, the definitive feature of an effector is that it is translocated into host cells. To test this, host cells infected for 18 h with wild type or *ssaR Salmonella* transformed with the HA tagged SseK3 expression plasmid were subjected to fractionation as described in the *Materials and Methods*. This procedure fractionates the infected host cells into a low speed pellet fraction (P) containing bacteria, unbroken cells, nuclei and large polymers, a high speed pellet (M) containing host cell membranes and a supernatant fraction (C) consisting of host cell cytosol. The lack of cross contamination between these fractions was demonstrated using antibodies against calnexin (host cell membrane protein), β tubulin (host cell cytosolic protein) and DnaK (Fig. 5). Both the M and C fractions are free of infecting bacteria and any bacterial protein in these fractions must have been transported from the bacteria into the host cell. For instance, the SPI2 effector control, SifA, can clearly be observed in the M fraction in cells infected with wild type, but not the SPI2 null *ssaR* strain (Fig. 5), demonstrating that SifA is translocated into host cells where it associates with host cell membranes as previously shown [23], [24]. SseK3 can be observed in both the M and C fractions in wild type infected cells (Fig. 5), demonstrating that it is delivered into host cells, consistent with being an effector. However, SseK3 expression could not be detected in the *ssaR Salmonella* infected cells and hence it cannot be concluded that translocation of SseK3 into host cells is dependent on T3SS-2.

### The role of SseK effectors in Salmonella virulence

**Figure 6:**
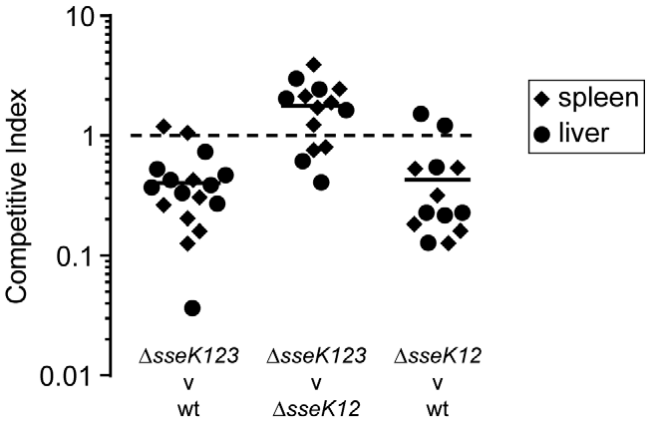
The role SseK effector in virulence. Competitive indices were determined 3 days following oral inoculation of 129/svImJ mice infected with an equal mixture of the indicated 2 strains. Values from spleen and liver for individual mice are shown as diamonds and circles respectively, and the mean is shown as a horizontal bar.

Previous work on SseK1 and 2 failed to find a role in virulence, either in terms of replication of *Salmonella* inside host cells or virulence in mice [12]. Considering that this could be due to functional redundancy with SseK3, which was functional in the strains tested by Kujat Choy *et al.* [12], we initially focussed our efforts in studying a strain mutated for all three SseK family members. Whilst we found no phenotype for the SseK family during intracellular replication in RAW264.7 cells (data not shown) we did identify a statistically significant (*P*<0.0001) attenuation at 72 h post-inoculation using competitive index infections of mice. (Fig. 6). This indicated that the SseK family of effectors do contribute to virulence. However, whilst a role in virulence for SseK1 and 2 could not be found in a previous study, we had concerns about the use of the time to death assay [12] because of it's lack of sensitivity and quantitation. Therefore we hesitated to conclude that SseK3 was responsible for the virulence defect, and investigated the role of SseK3 further. Competitive index experiments comparing the virulence of an *sseK3* strain to wt indicated no role SseK3 in virulence (Fig. 6), suggesting that the results we had obtained with the *sseK123* triple mutant were most likely due to SseK1 and/or 2. We confirmed this hypothesis by comparing an *sseK12* double mutant to wt in competitive index experiments and found a statistically significant degree of attenuation (*P* = 0.0007), similar in magnitude to that of the *sseK123* triple mutant. So it is clear that a minor role in virulence for the SseK family exists, it seems unlikely that SseK3 itself plays a significant part.

## Discussion

Above we identified and described a member of the SseK/NleB family of effectors that was encoded in phage ST64B by genome comparison between the virulent SL1344 strain of *S. typhimurium* and the laboratory strain LT2. This new member, SseK3, has a somewhat limited distribution in the available *Salmonella* genomes (Table 1), consistent with it being encoded on a temperate phage. In contrast, SseK1 and SseK2, which are not phage-encoded, are present in most available *Salmonella* genome sequences.

**Table 1:** Strain distribution of phage encoded effectors of *Salmonella*.

Serotype (strain)	SseK3	SopE	SspH1	GogB	SseI
4,[5],12:i:- (CVM23701)	+	−	−	+	+
Agona (SL483)	−	−	−	−	−
Choleraesuis (SC-B67)	−	−	−	+	+
Dublin (CT_02021853)	+	+	−	−	+
Enteritidis (PT4)	+	+	−	−	+
Gallinarum (287/91)	−	+	−	−	−
Hadar	−	+	−	−	−
Heidelberg (SL476)	−	+	−	−	−
Heidelberg (SL486)	−	+	−	−	−
Infantis	−	−	−	−	−
Javiana (GA_MM04042433)	−	+	+	−	−
Kentucky (CDC 191)	−	−	−	−	−
Kentucky (CVM29188)	−	−	−	−	−
Newport (SL254)	−	−	−	+	+
Newport (SL317)	−	−	+	+	+
Paratyphi A (ATCC9150)	−	+	−	−	−
Saintpaul (SARA23)	−	−	+	−	−
Saintpaul (SARA29)	−	+	+	−	−
Schwarzengrund (CVM19633)	−	+	−	−	−
Schwarzengrund (SL480)	−	+	−	−	−
Typhi (CT18)	−	+	−	−	−
Typhi (Ty2)	−	+	−	−	−
Typhimurium (D23580)	+	−	−	+	+
Typhimurium (DT104)	−	−	−	+	+
Typhimurium (DT2)	+	−	−	+	+
Typhimurium (LT2)	−	−	−	+	+
Typhimurium (14028s)	+	−	+	+	+
Typhimurium (SL1344)	+	+	−	+	+

SseK3 expression was induced, albeit to a relatively low level, in SPI2 inducing minimal medium and inside host cells, whereas no expression was observed under SPI1 inducing conditions. This expression profile led us to show that SseK3 is translocated into host cells following intracellular growth of *Salmonella*. However, the relatively low level of SseK3 expression combined with the low intracellular growth rate of T3SS-2 null *Salmonella* precluded effective assessment of the role of T3SS-2 in translocation of SseK3. Similarly, secretion of SseK3 into culture supernatants could be detected in the hypersecreting *ssaL* strain [22] but not the wild type strain, again possibly due to relatively low levels of expression. The phenotype of the *ssaL* strain, and the demonstrated role of SsaL in substrate recognition for T3SS-2 secretion [22] do provide some support the hypothesis that SseK3 is a T3SS-2 translocated effector. Quantitative virulence experiments in mice indicated no role for SseK3 in virulence, though, contrary to a previous report [12] we did find evidence to implicate SseK1 and/or SseK2 in *Salmonella* virulence. On balance, we suggest that SseK3 is an effector that is expressed in small quantities, has a limited role in virulence, and remains to be defined in terms of its secretion and phenotype.

The approach used to identify SseK3 focused on identifying type III secreted effectors in strain SL1344 that are homologous to other effectors and absent from strain LT2. This approach was validated by identifying the effector SopE, and further describing its genomic context in SL1344 relative to LT2. There are aspects to this approach that, with modification, may lead to the identification of more effectors. The criteria that genes must show similarity to known effectors could be disregarded in order to produce a longer list of low GC content genes to characterise, some of which would be likely to encode novel effectors. Another potential modification is to search for differences between more *S. typhimurium* strains, which may harbour different complements of effectors. Extending this analysis to different serovars within the genus *Salmonella* is likely to provide and even more complete list of *Salmonella* effectors. The larger number of horizontally acquired genomic differences between serovars has previously been identified and shown to encode functions involved in virulence that remain otherwise uncharacterised [25].

The identification of a new effector in *Salmonella* raises the possibility that more effectors remain to be identified. The role in virulence played by the majority of effectors is minimal [26]–[29]. Similarly, most T3SS dependent phenotypes are not understood in terms of the effectors involved, with invasion and intercellular junction disruption being exceptions [30], [31]. Hence it is clear that our understanding of effector repertoire and function is far from complete. In considering these points together with the fact that attaching and effacing pathogens employ one T3SS to translocate up to 49 effectors in order to parasitise the gastrointestinal tract [32], it seems possible that *Salmonella*, using two T3SSs to parasitise the gastrointestinal tract and systemic sites, will encode numerous effectors in addition to the 28 described thus far. Characterisation of these is necessary to understand the pathogenesis of *Salmonella* infection.

The identification of SseK3 brings the number of phage-encoded effectors in SL1344 up to four (SopE, SseI and GogB) [33], with SspH1 being found in *S. typhiumurium* strain 14028 [34]. Of these, GogB is expressed under a broad range of culture conditions and when transferred into attaching and effacing *E. coli* is expressed and secreted by the locus of enterocyte effacement encoded T3SS [33]. SspH1 has a similar expression pattern to GogB [16], [34], and may also have the capacity to be secreted by heterologous T3SSs. This is unlikely to be the case for SseK3 or SseI, which appear to be specifically controlled by the SPI2-encoded SsrA/B regulatory system. For instance SseK3 is not expressed in *E. coli* DH5α (Fig. 2A).

The distribution of these phage-encoded effectors (Table 1) in the available *Salmonella* genome sequences, determined as described in the Materials and Methods, demonstrates the plasticity of the effector complement across a range of strains. The SseK3 encoding phage, ST64B, has also been shown to be present in *S. typhimurium* strain 14028 and three strains of DT104 [8], yet it is not found in the DT104 strain whose genome has been sequenced at the Sanger Institute. The variable presence or absence of *sseK3* in DT104, an *S. typhimurium* phage-type considered to be essentially clonal [35], affirms that *Salmonella*-host interactions are currently evolving. SseK3, GogB and SseI are relatively more abundant in *S. typhimurium* than other serovars. On the other hand, non-Typhimurium serovars seem to have a more variable content of phage-encoded effectors, with SopE being particularly prevalent. Reasons for this most likely reflect a combination of the research bias towards *S. typhimurium* as well as differences in virulence and host range among the surveyed serovars. Horizontal acquisition of SopEϕ phage has been shown into increase virulence in a SopE dependent manner [36]. This, together with recent data from an attaching and effacing pathogen-host system demonstrating that virulence mediated by type III effectors is positively selected [37], suggests that reshaping effector content by horizontal gene transfer is linked to evolutionary success. This, of course, is a challenging hypothesis that remains to be tested, and also underscores the requirement to study a larger sample of *Salmonella* serovars in order to identify all effectors and understand their contributions to *Salmonella* virulence.

In summary, SseK3 is a phage-encoded effector that belongs to the SPI2 regulon and is translocated into host cells. Together with the other phage-encoded effectors of *Salmonella*, SseK3 likely represents the cutting edge of the evolution of *Salmonella*-host interactions.

## Materials and Methods

### Ethics statement

All experiments with animals were conducted according to guidelines set by the Canadian Council on Animal Care. The protocol (A09-0168) used during this work was approved by the University of British Columbia's Animal Care Committee.

### Bioinformatics

The completed draft SL1344 chromosome sequence was obtained from the Sanger Institute website (http://www.sanger.ac.uk/Projects/Salmonella/SL1344.fasta) and the LT2 chromosome sequence was obtained from Genbank (accession # AE006468). These sequences were compared using DOUBLE ACT V2 (http://www.hpa-bioinfotools.org.uk/pise/double_act.html#) with a cutoff score of 1000 to prepare a MEGABLAST [38] output for analysis in ARTEMIS COMPARISON TOOL V6 [39]. Using the preliminary gene prediction provided by the Sanger Institute, we performed similarity searches of the deduced amino acid sequences of predicted SL1344 genes not present in LT2 using standalone BLAST V2.2.17 [40]. These searches were performed with default settings against the non-redundant database (nr) release from the 15th of October, 2007.

Amino acid sequence alignments were performed using CLUSTAL W [41] and shaded and formatted using JALVIEW v8.0 [42]. The survey of phage-encoded effectors among *Salmonella* genomes was done by performing TBLASTX [40] searches of all *Salmonella* genomes available at the NCBI genomic website (http://www.ncbi.nlm.nih.gov/sutils/genom_table.cgi) and the Sanger Institute website (http://www.sanger.ac.uk/cgi-bin/blast/submitblast/salmonella). A strain was considered to have a copy of a given effector if it had >90% identity over the full length of each sequence.

### Bacterial strains, plasmids and culture

*Escherichia coli* strain DH5α was used for routine cloning and *S. typhimurium* strain SL1344 and isogenic mutants thereof, referred to by genotype, were used in this study. pSseK3-HA, the C-terminally HA epitope tagged SseK3 expression plasmid, was constructed by replacing the *Sal* I-*Bgl* II fragment containing *gogB* and its upstream region of pWSK129-*gogB*-2HA [33] with a PCR product containing *sseK3* and its upstream region. The PCR product was generated using primers sb26f2 (5′-ATGGTCGACCACAGCAATTAATCTTCTGC-3′) and sb26r (5′-ATGAGATCTTCTCCAGGAGCTGATAGT-3′). To create an ampicillin resistant version of this plasmid, termed pSseK3-HA.1, the *Sal* I-*Xba* I fragment containing *sseK3* was cloned into the corresponding sites of pWSK29 [43]. A plasmid expressing the *sseK3* promoter fused to *gfp* was constructed as per Coombes *et al.* [44] and p*sifA*-2HA and p*pipB*-2HA has been described previously [19], [24]. Lysogeny broth [45], [46] was used for the routine culture of all bacteria. SPI1 induced, invasive *Salmonella* were cultured in LB as previously described [47] and SPI2 induced *Salmonella* were grown to logarithmic phase with shaking at 37°C in LPM medium [15].

### Basic biochemistry techniques

For expression analysis of SseK3, bacteria from 1 ml of culture grown in the indicated media were harvested by centrifugation at 16,000 g for 1 minute, the supernatant discarded and the pellet dissolved in 200 µl of 1x Laemmli sample buffer [48]. For *in vitro* secretion assays, culture supernatants were passed through a 0.2 µm syringe filter and the proteins precipitated by the addition of trichloroacetic acid to a final concentration of 10% (v/v) in a total volume of 2.0 ml. This was incubated on ice overnight and the precipitated protein collected by centrifugation at 16,000 g for 1 h at 4°C. The supernatant was discarded and the pellet washed in ice-cold 100% (v/v) acetone, followed by centrifugation at 16,000 g for 15 min at 4°C. The supernatant was discarded and the pellet dissolved in 20 µl of 1x Laemmli sample buffer.

HeLa cells were infected as previously described [47] and fractionation was performed according to the protocol described by Gauthier *et al.* [49]. At 18 h postinfection, cells were collected by scraping, lysed by passage through a 22-gauge needle and fractionated into fraction P (a low speed pellet containing nuclei, unbroken cells, bacteria and large polymers), fraction M (a high-speed pellet containing host cell membranes) and fraction C (the supernatant from a high speed centrifugation corresponding to host cell cytosol). All fractions were dissolved in 1x Laemmli sample buffer.

The mouse anti-HA antibody (Covance) was used at a dilution of 1∶2000, the mouse anti-DnaK antibody (Stressgen) was used at a dilution of 1∶2000 and the anti-calnexin antibody (Stressgen) was used at a dilution of 1∶5000. The anti-β-tubulin antibody (E7) was used at a dilution of 1∶1000 and obtained from the Developmental Studies Hybridoma Bank, developed under the auspices of the NICHD, National Institutes of Health, and maintained by the University of Iowa (Department of Biological Sciences, Ames, IA, USA).

### Flow cytometry

Primary bone-marrow derived macrophages (BMDMs) were derived as previously described [50]. At 30 min and 8 h postinfection, supernatants were removed from macrophages were washed three times in sterile PBS. Cells were scraped into 300 µl of cold fluorescence-activated cell sorting (FACS) buffer containing PBS, 2% FBS, 0.1% sodium azide, and 1 mM EDTA and transferred into a 96-well round-bottomed tissue culture plate. Plates were centrifuged for 3 min to pellet cells, and FACS buffer was removed. RAW macrophages were incubated with 25 µl of biotinylated primary antibody F-480 (Serotec, Raleigh, NC) at a dilution of 1∶300 in FACS buffer for 30 min at 4°C. Cells were washed twice in FACS buffer followed by incubation with streptavidin-phytoerythrin (Cedarlane Laboratories Ltd., Hornby, ON, Canada) at a dilution of 1∶400 in FACS buffer for 30 min at 4°C. Cells were washed twice in FACS buffer and suspended in a final volume of 150 µl of FACS buffer for flow cytometry analysis. Samples were run on a FACSCalibur flow cytometer, with data for 10,000 events collected per sample using Cell Quest Pro software (BD Biosciences, Mississauga, ON, Canada). Data were analyzed using Flow Jo flow cytometry software (Tree Star Inc., Ashland, OR).

### Infection of mice

Mice were infected as previously described [44]. Briefly, mice were inoculated orally with approximately 5×10^7^ of each strain, one of which was marked with chloramphenicol resistance (*ushA*::*cat*, which shows wild type virulence [14], [51]). At 72 h post-inoculation, mice were killed and the spleens and livers were removed and homogenized. Organ homogenates were serially diluted and plated on L-agar plates containing streptomycin for determination of total numbers of *Salmonella*. Plates were replica plated onto L-agar plates containing chloramphenicol to determine the proportion of *ushA*::*cat Salmonella* present in the homogenate, allowing determination of competitive indices as previously described [52], [53]. Data was analyzed using a one-sample *t* test against a theoretical mean of 1.
